# Surgical Treatment of Liver Metastases in Neuroendocrine Neoplasms

**DOI:** 10.1155/2012/782672

**Published:** 2012-01-26

**Authors:** Palepu Jagannath, Deepak Chhabra, Shailesh Shrikhande, Rajiv Shah

**Affiliations:** ^1^Department of Surgical Oncology, Lilavati Hospital & Research Centre, Mumbai 400 050, India; ^2^Department of Surgical Oncology, Dr. L. H. Hiranandani Hospital and Research Centre, Mumbai 400 076, India; ^3^Department of Gastrointestinal Surgical Oncology, Tata Memorial Hospital, Mumbai 400 012, India

## Abstract

Neuroendocrine neoplasms (NENs) are a distinctive entity, and nearly 10% of patients already have liver metastases at presentation. The management of neuroendocrine liver metastases (NEN-LM) is complex with differing patterns of metastatic presentation. An aggressive approach should be used to resect the primary tumor, to remove regional lymph nodes, and to resect or treat appropriate distant metastases (including liver tumors). Despite having an indolent course, NENs have a significantly reduced survival when liver metastases are untreated. Though a wide range of therapies are now available with a multimodal approach to the treatment, surgical treatment offers the only chance for a significant survival prolongation and/or improvement of symptoms and quality of life. A review of the existing surgical modalities for NEN-LM is discussed in this paper.

## 1. Introduction

Neuroendocrine tumors (NETs) consist of a group of neoplasms that arise from neuroendocrine cells dispersed throughout the body and show variable clinical course. The World Health Organization (WHO) classifications in 2000 and subsequently in 2004 did not address the diversity of these tumors. A histologic grading system based on Ki-67 labelling index was proposed by the European Neuroendocrine Tumour Society (ENETS) [1, 2]. The ENETS grading system (G1, G2, G3) has thus been incorporated in the new WHO 2010 classification [3]. It is now recognized that all neuroendocrine tumours are potentially malignant and hence characterized as neuroendocrine neoplasms (NENs). Intestinal NENs represent two-thirds [4], while pancreatic NENs represent about one-third of gastroenteropancreatic NENs (GEP-NENs) [5]. Besides regional lymph node involvement, liver is the predominant site of metastases [6]. Up to 75% of patients with small bowel NEN and 30–85% of pancreatic NENs present with liver metastases (NEN-LM) either at initial evaluation or during the course of their disease [7–9]. An additional 5–10% of NEN patients present with liver metastases with unknown primary tumor site.

In contrast with the traditional opinion that NEN represents an indolent disease, Touzios et al. [10] reported 5-year survivals range from 13–54% in patients with untreated NEN-LM compared to 75–99% in those without liver metastases [11–15].

## 2. Liver Metastases as a Prognostic Factor

Pancreatic NENs have a lower 5-year survival rate (30–60%) compared to intestinal NENs (60–90%) [16–18]. Liver metastases, however, are the most important prognosticator of survival in patients with NEN regardless of the primary site [19].

Two large population-based studies [7, 20] with 13715 and 4104 patients, reported that 12.9% of patients already had liver metastases at initial diagnosis regardless of tumor location and 5–10% of patients had metastases with unknown primary. Occasionally a primary neoplasm is not found elsewhere despite extensive investigations, raising the possibility that the hepatic lesion is the primary tumour [21]. This might be due to the low sensitivity of currently available imaging techniques, although this seems increasingly less likely with advances in technology such as helical computed tomography (CT), endoscopic ultrasonography (EUS), and Gallium-68 PET CT.

Histological subtypes have an influence on treatment and survival outcomes. The reported overall survival ranges from 5.2 to 57% with different histological subsets of digestive NETs [7, 20]. A 95% survival at 20 years has been reported for patients with gastrinoma without liver metastases in contrast with 15% 10-year survival in the presence of bilobar hepatic metastases [22]. 5-year survivals of midgut and hindgut NET decrease by 10–20% and 50–60%, respectively, in the presence of liver metastases [23–26]. The new WHO classification (2010) emphasizes the importance of grades G1–3. Tumors with <2 mitosis/10 hpf and <3% Ki67 index are well differentiated and are labelled as G1 tumors, while well-differentiated tumors with 2–20 mitosis/10 hpf or 3%–20% Ki67 index are designated as G2. High immunohistochemical expression of Ki67 is a strong marker of poorly differentiated NETs, and tumors with >20 mitoses/10 hpf or a Ki67 >20% are labelled as G3 tumors [3]. Well-differentiated G1 tumors tend to be more indolent and are good candidates for liver-directed therapy, whereas poorly differentiated G3 neuroendocrine carcinomas (with or without liver metastases) are highly aggressive and patients (even with treated metastatic disease) have an expected survival time of 6–18 months [27, 28]. These tumors are not proposed for surgical resection and are usually confined to systemic chemotherapy (commonly Cisplatin and Etoposide combination).

## 3. Distribution of Hepatic Metastases

The pattern of distribution of liver metastases is an important determinant of prognosis [26, 29, 30]. Three different patterns of NEN-LM are identified that have an impact on the therapeutic approach: Type I: “restricted metastases,” that is, the metastases are confined to one liver lobe or limited to two adjacent segments. This pattern is usually seen in 20–25% of the cases; the metastases are clearly resectable and can be dealt with by a standard anatomical resection; Type II: “dominant lesion with bilobar metastases” in which there is one dominant lesion but with smaller satellites contralaterally. Such bilobar patterns occur in 10–15% of the cases; the metastases may be potentially resectable and can still be approached surgically with a combination of ablative therapy on the contralateral lobe; Type III: “diffuse, multifocal liver metastases” are found in 60–70% of the cases and surgery is not a good option for these tumors [31, 32]. Type III tumors are clearly unresectable, and a cautious option of liver transplant may be considered for these tumors. Thus, the extent of hepatic involvement of metastatic NEN limits the benefit of surgery in a substantial majority of patients and standard resection alone is inadequate [33]. Nevertheless, it is evident that Type I NEN-LM are associated with favourable outcomes compared to the other two types [10, 30].

## 4. Diagnostic Work up for Neuroendocrine Liver Metastases

Combined anatomic and functional imaging studies provide tumor localization and assessment of posttreatment outcomes. Our current practice of evaluation is a Triphasic Triplanar CT scan with 1-2 mm slice thickness. A typical contrast enhancement in the arterial phase of the scans is characteristic due to the hypervascular nature of these tumors. However, depending on the tumor type, size, and location, the portal and parenchymal phases of contrast enhancement may also be important for improved detection [34–37].

Magnetic resonance (MR) imaging is complimentary and especially helpful in patients unable to receive iodinated contrast agents. One study [38] showed that MR imaging can detect more liver lesions, and a T2-weighted imaging may detect most lesions when contrast agents cannot be given.

Somatostatin receptor scintigraphy (SRS) has rapidly evolved as the gold-standard imaging procedure for NEN expressing somatostatin receptor subtype 2. Indium-labelled somatostatin analogues have been replaced by Gallium-labelled analogues that in combination with a PET-CT (68 Ga-DOTATOC PET/CT) increase the diagnostic sensitivity up to 30% higher than the conventional scanning. Moreover, SRS has resulted in a change in the clinical management in 33–77% of NEN patients in various studies [31, 39].

Beside the advantage of total-body imaging with the potential of simultaneous visualization of the primary tumour and metastatic deposits, SRS can possibly identify those patients who might be candidates for somatostatin receptor-based radiotherapy [39–41].

Plasma chromogranin A (CgA) is a widely accepted tumour marker with respect to diagnosis, prognosis, and monitoring of the treatment [42–45]. Though the sensitivity of CgA depends upon the NEN type and tumour burden, patients with NEN-LM tend to have significantly higher CgA concentrations than those without metastases [46]. Additional assessment of insulin, C-peptide, gastrin, pancreatic polypeptide, vasoactive intestinal peptide, glucagon, calcitonin, and somatostatin should be useful depending on the tumor functional status, clinical symptoms, and histological features.

A core needle biopsy and a histological examination with immunohistochemistry (IHC), Ki-67, and mitotic index of the primary/metastasis is essential for planning treatment. Tumour staging predicts the prognosis and tailors the therapeutic strategy [32, 47] particularly in patients who are not candidates for complete resection.

## 5. Liver-Directed Therapy

No optimal therapeutic strategies exist for treatment of liver metastases from GEP-NEN, and best strategy for treatment of NEN-LM is still poorly defined [48, 49]. Moreover, there is no randomized trial comparing surgery with nonsurgical treatments like RFA (radiofrequency ablation), TACE (transarterial chemoembolization), and medical treatment. In view of the infrequency of these tumours, multicentre clinical trials are needed in addressing the role of surgery.

### 5.1. Resection

#### 5.1.1. Does Resection Benefit?

Surgery is generally proposed to all patients with operable well-differentiated metastases from digestive NENs regardless of the site of origin [32]. However, most NENs are detected after extensive liver metastases are present, and, consequently, only 10% to 20% of patients with NEN-LM are eligible for resection [50, 51].

The benefits of surgical resection for NEN-LM have been demonstrated in terms of overall survival and quality of life. Overall survival after hepatic resection has been reported in 46–86% at 5 years and 35–79% at 10 years in various series [52]. Complete resection (R0/R1) for both mid- and hindgut tumors is associated with better long-term survival [30, 53–56]. In many reported series of patients in whom hepatic resection was feasible, a median survival time was not reached during a followup of 27 months [56] up to 78 months [57] compared with 27 months [56] and 17 months [57] in those with unresectable tumours.

A recent multicenter study evaluating 339 patients who underwent surgical management of NEN-LM from 1985 to 2009 identified those who are likely to benefit the most by liver-directed surgery. It was observed that patients with hormonally functional NEN who had R0/R1 resection benefited the most from surgery [58]. Another large study [59] observed that R1 resections, unlike many other cancers, were not associated with a worse overall survival after liver resection for NEN-LM.

Resection is associated with a low mortality rate (0–5%) and an acceptable morbidity (close to 30%), and up to 95% of patients have shown symptom improvement in one large surgical series of 170 patients [56].

R0 resection rates have been reportedly between 20 and 57% in various series [31, 55, 56, 60–62]; however, among patients undergoing complete resection, long-term disease-free survival is reported in up to only 20 percent of patients [53, 63].

Such variability of clinical outcomes demands a meticulous case selection, and certain prerequisites should be considered prior to a resectional surgery [32, 52, 64]: (i) resectable primary tumor (previously resected or considered resectable synchronously), (ii) well-differentiated NEN-LM, (iii) possibility of R0 resection, (iv) exclusion of nonresectable extrahepatic disease, (v) reasonable performance status, and (vi) corrected or optimised carcinoid heart disease prior to aggressive liver surgery.

 The presence of local recurrence including abdominal lymph node involvement is not an absolute contraindication for surgery if the removal of liver metastases and lymph nodes and/or the recurrence site(s) is planned [32].

In all cases in which the patients have carcinoid syndrome, specific perioperative treatments with somatostatin analogues are indicated to prevent intra- and postoperative carcinoid crisis [65, 66].

#### 5.1.2. Recurrence after Resection and Impact of R0 Resection

Recurrence after an R0 resection is not uncommon, and 5-year local recurrence rates of up to 97% have been reported even when complete resection has been achieved [53, 55, 67, 68]. Recurrence depends mainly on the initial completeness of liver resection, and a thorough pre- and intraoperative assessment of small liver metastases is essential.

In a large series of 170 surgically treated patients, 5- and 10-year recurrence rate was 84% and 94%, respectively, with a median time to recurrence of 21 months. Only 44% of patients had a complete tumour resection in this series with a 5-year recurrence rate of 76% and a median time to recurrence of 30 months. In comparison patients who did not undergo a complete resection showed a 5-year recurrence rate of 91% with a median time to recurrence of only 16 months [53].

The prognostic relevance of R0 resection has been pointed out by Gomez et al. in their report of 18 resected patients who showed an overall 5-year recurrence rate of 34%. The five-year recurrence was only 10% in patients with tumour-free resection margins, in contrast to 75% when resection margins were involved [68]. Thus, an aggressive surgical approach does benefit irrespective of completeness or R0 status and has an impact on prognosis.

#### 5.1.3. Resection Strategies in Synchronous and Metachronous Tumors

Unlike most malignancies, resection of the primary is beneficial for patients with NENs and should be considered in patients who have resectable metastatic disease [69, 70]. However, resection of a small asymptomatic (relatively stable) primary in the presence of unresectable metastatic disease is not indicated [69].

In synchronous disease, liver surgery can be performed either as a one-step or a two-step procedure [32, 55, 71]. NEN-LM may be resected at the same time as the primary tumor with little additional risk if the metastases are unilobar [54, 55]. The main consideration however should be to perform a complete resection with acceptable morbidity rate. If major or complex liver resection is required, a two-stage surgery may be preferable in order to reduce the operative risk especially in patients with Type II metastases. A two-step surgery may involve at the first step a resection of metastases of the one lobe in addition to a resection of the primary and lymph nodes. Contralateral liver volume enhancement by portal venous embolization is an option with an aim to induce left liver hypertrophy followed by right hepatectomy or Lobectomy as a second step. Such an approach can in selected patients avoid or delay indications for liver transplantation [71]. For patients with unresectable liver metastases, a cholecystectomy is recommended to prevent ischemic complications of the gallbladder subsequent to chemoembolization and possible gallstones formation during somatostatin analogue therapy [54].

For metachronous liver metastases, a one-step procedure can be recommended as a low-risk approach to unilobar disease (<30% morbidity). For bilobar or diffuse liver metastases, a sequential approach including resection with or without ablative techniques, preoperative portal embolization, percutaneous treatments, or intra-arterial chemoembolization may be adopted [32, 72].

Overall, the effectiveness of the resection of unilobar and bilobar liver metastases depends on the operative techniques employed as well as the competence of the hepatobiliary surgeon. Intraoperative ultrasonography is essential in defining the extent of any known lesions and to detect any additional smaller lesions missed during a preoperative diagnosis. Resectional surgery should be the first option before patient is considered for liver transplantation due to standard priority in listing.

#### 5.1.4. Does Debulking Benefit?

Several retrospective series have suggested that selected patients who undergo aggressive “debulking” of NEN-LM, in which the majority but not all of the disease is resected, have better quality of life and longer survival relative to those who do not undergo surgery [10, 30, 73–77]. Soreide et al. [78] found that patients with NEN hepatic metastases who underwent surgical debulking (planned repeat operations included) had a three- to fourfold longer median survival time compared with those who did not. However, complication and mortality rates were high (33% and 9%, resp.), and the duration of symptom relief in most cases was 6–24 months.

Incomplete debulking surgery (R2) has limited indications, yet it can improve the quality of life in selected patients for whom medical treatment has failed. However, in order to be efficient, the removal of at least 90% of the tumor volume is required [54, 56, 79, 80].

Thus, when complete resection of NEN-LM is not feasible or in the presence of unresectable extrahepatic disease, a tumor debulking strategy should be considered especially in patients with functional NENs with hormonal symptoms refractory to other treatments. Debulking can be a strategy for nonfunctioning NENs with local effects such as abutting the hepatic hilum (resulting in biliary obstruction) or obstructing the colon/duodenum [47, 81].

A combination of techniques, namely, resection and ablation or resection combined with other liver-directed therapy should be used to achieve complete tumor response when all liver disease cannot be resected.

### 5.2. Local Ablative Techniques

Radiofrequency ablation (RFA) has become the preferred local-ablative therapy in most centres, and its use has been shown to be effective in both relieving the symptoms of NEN-LM and achieving local control of the metastases [32, 82, 83].

Mazzaglia and colleagues reported the largest experience of ablation in patients with NEN-LM, encompassing a total of 452 lesions in 63 patients via 80 laparoscopic RFA sessions. Thirty-six patients were symptomatic from disease, and 94% experienced symptom relief after ablation for a median duration of 11 ± 2.3 months after RFA. The procedure-associated morbidity was 5%, and there was no 30-day mortality. Median survival was 3.9 years calculated from the first RFA session with a 2-year survival of 77% [84].

In yet another study of patients with 234 NEN metastases, 34 were treated with RFA. 80% of the patients reported a complete or significant relief from their symptoms, lasting for an average of 10 months and 41% of the treated patients showed no evidence of progression [85].

Tumor size poses a significant limit on the effectiveness of RFA. Though ablation may be used repeatedly within the same metastasis, it is difficult to fully eradicate with certainty tumors that are >3 cm in diameter, and a tumor >5 cm in diameter is considered to be unsuitable for RFA [86].

RFA has been shown to be a relatively low-risk procedure for treating liver tumors [87], and while the safety of RFA makes it an attractive method of treatment, the rate of tumor recurrence after therapy limits its effectiveness as a single therapy [86]. A recent study reported progressive liver disease in 80% of patients with NEN liver metastases treated with RFA [84].

### 5.3. Combination Techniques of Resection and Other Modalities

#### 5.3.1. Resection Combined with Cryoablation

While liver resection for NEN-LMs provides the best chance of long-term survival, it is unfortunately not feasible in the majority of patients given the often widespread presentation of liver disease. Combining resection with local ablation can potentially expand the resection criteria and thus help improve survival [88]. In a recent study, forty patients with NEN-LMs underwent concomitant hepatic resection and cryoablation between 1992 and 2010 with a median followup of 61 months (for alive patients). The median progression-free survival and overall survival after hepatic resection were 22 and 95 months, respectively. Five-year and 10-year overall survival rate was 61% and 40%, respectively. While histologic grade was an independent factor associated with overall survival, presence of extrahepatic disease was associated with progression-free survival.

It thus appears that concomitant hepatic resection and cryoablation to achieve tumor debulking is associated with good survival outcomes in well-selected patients. This recent report suggested that such an approach may increase the number of patients with borderline resectable disease undergoing surgical management of advanced NEN-LMs [88].

#### 5.3.2. Resection and Radiofrequency Ablation

Therapy with RFA alone is associated with higher recurrence rates compared to RFA plus resection, and in patients whose metastases are otherwise unresectable or difficult to access, the combination of resection and RFA provides the opportunity to achieve complete tumor removal [89–91].

Elias et al. [92] reported an overall survival rate of 84% at 3 years by incorporating a one-step combined approach of hepatectomy (for large or contiguous NEN-LMs) along with intraoperative use of multiple RFAs (for remnant metastases <2.5 cm). A mean of 15 ± 9 NEN-LMs per patient were surgically removed, and a mean of 12 ± 8 (median of 10) NEN-LMs per patient were RF ablated.

A combination of RFA along with parenchyma preserving liver resections seems to be the way forward while dealing with multiple bilobar liver metastases that are unlikely to be completely resected by surgery alone.

#### 5.3.3. Resection and Chemoembolization

Chemoembolization is indicated for nonresectable multiple bilobar metastases, and in various studies 55%–100% of patients with malignant NENs treated by hepatic arterial embolization (HAE)/transarterial chemoembolization (TACE) have symptomatic improvement and 20%–80% have an objective response with tumor shrinkage. The mean duration of response ranges from 6 to 42 months [93–96].

Advances in major liver resectional surgery has resulted in further development of multimodal approaches for NELMs where surgeons and interventional radiologists have tried to work in multidisciplinary settings to evaluate whether TACE and surgery can have a synergistic action on overall outcomes of NELMs. Hepatic resection may be possible after cytoreduction of the tumor following TACE and other therapies [97]; however, the data on this subject is sparse.

### 5.4. Liver Transplantation

In patients with diffuse unresectable liver metastases or who suffer from life-threatening hormonal disturbances refractory to medical therapy, liver transplantation may be an option for carefully selected patients [32].

Primary tumor location has an impact on outcomes of liver transplantation. While the 5-year survival rate was 68% in patients with limited hepatic disease and non-duodenopancreatic tumours, it dropped to 12% in the case of hepatomegaly and primary tumour localized within the duodenum or pancreas [98, 99].

Majority of patients undergoing orthotopic liver transplantation (OLT) ultimately develop recurrent disease and reported 5-year recurrence-free survival ranges from 24 to 45% with an overall survival range of 36–57% [100–105].

Mazzaferro et al. could achieve a 90% overall survival and a 77% recurrence-free survival at 5 years by defining specific criteria for indication of liver transplant in the setting of NLM: (a) well-differentiated NENs (low-grade functioning or nonfunctioning), (b) a prior curatively resected primary tumor drained by the portal system, (c) ≤50% metastatic involvement of the liver, (d) good response or stable disease for a minimum of 6 months prior to transplantation, and (e) age ≤ 50 years [106].

An early disease recurrence, a considerable postoperative mortality, the absence of extensive experience, and lack of universal indications have precluded orthotopic liver transplantation as a good option for most patients with unresectable NEN-LMs [107]. Moreover, limited availability of donor organs in many regions has been a barrier to the widespread use of liver transplantation in general. Thus, the potential benefit of liver transplantation in patients with malignant NENs needs to be weighed against issues of perioperative morbidity and the ethical distribution of donor organs [32].

**Figure 1:**
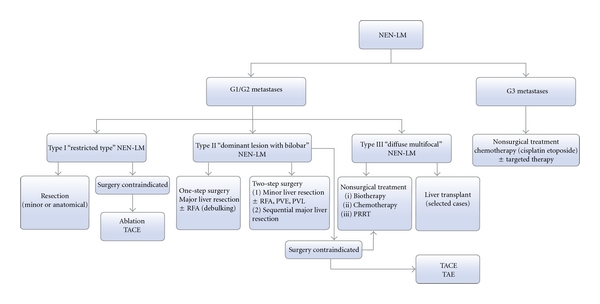
Suggested treatment algorithm for patients with NEN-LM. NEN: neuroendocrine neoplasm; LM: liver metastasis; RFA: radiofrequency ablation; TACE: transcatheter arterial chemoembolization; TAE: transcatheter arterial embolization; PVE: portal vein embolization; PVL: portal vein ligation.

A modified algorithm for the treatment of patients with metastatic NETs based on ENETS consensus guidelines [32] is shown in Figure 1.

## 6. Summary

Surgical resection remains the gold standard especially in the treatment of well-differentiated NEN-LMs for symptom relief and long-term survival. In both synchronous and metachronous tumors, one- and two-step procedures may be undertaken, depending upon whether the liver disease is unilobar or complex.

Debulking resections are justified in functioning NEN and selective nonfunctioning NENs; however, removal of at least 90% of the tumor volume is necessary.

RFA can be used effectively as antitumor treatment and as a sole therapy for relieving symptoms in patients with NEN-LMs, but when combined with resection a better outcome is anticipated.

Liver transplantation needs to be carefully considered in specific liver alone bilobar metastases especially in (low-grade) well-differentiated NENs.

Surgical options are complimented by ablative techniques (RFA/cryoablation), nonsurgical liver-directed therapies (HAE/TACE/Transarterial radioembolization—TARE), and systemic treatment modalities (peptide receptor radiotherapy, cytotoxic chemotherapy, somatostatin analogues, and newer molecular-targeted treatments). A multidisciplinary team approach is necessary to customize therapy for each patient with NEN-LM.
